# Ventilator-Associated Pneumonia and PaO_2_/F_I_O_2_ Diagnostic Accuracy: Changing the Paradigm?

**DOI:** 10.3390/jcm8081217

**Published:** 2019-08-14

**Authors:** Miquel Ferrer, Telma Sequeira, Catia Cilloniz, Cristina Dominedo, Gianluigi Li Bassi, Ignacio Martin-Loeches, Antoni Torres

**Affiliations:** 1 Department of Pneumology, Hospital Clinic of Barcelona, August Pi i Sunyer Biomedical Research Institute—IDIBAPS, University of Barcelona, 08036 Barcelona, Spain; 2 Network Centre for Biomedical Research in Respiratory Diseases (*CibeRes*, CB06/06/0028), Carlos III Health Institute, 28029 Madrid, Spain; 3 Department of Pneumology, Hospital Prof. Doutor Fernando Fonseca, EPE, School of Medicine, University of Lisbon, 2720-276 Lisbon, Portugal; 4 Department of Anesthesiology and Intensive Care Medicine, Fondazione Policlinico Universitario A. Gemelli, Sacred Heart Catholic University, 00168 Rome, Italy; 5 St. James’s Hospital, Multidisciplinary Intensive Care Research Organization (MICRO), Dublin D03 VX82, Ireland

**Keywords:** intensive care unit, ventilator-associated pneumonia, nosocomial infection, PaO_2_/F_I_O_2_

## Abstract

Background: Ventilator-associated pneumonia (VAP) is associated to longer stay and poor outcomes. Lacking definitive diagnostic criteria, worsening gas exchange assessed by PaO_2_/F_I_O_2_ ≤ 240 in mmHg has been proposed as one of the diagnostic criteria for VAP. We aim to assess the adequacy of PaO_2_/F_I_O_2_ ≤ 240 to diagnose VAP. Methods: Prospective observational study in 255 consecutive patients with suspected VAP, clustered according to PaO_2_/F_I_O_2_ ≤ 240 vs. > 240 at pneumonia onset. The primary analysis was the association between PaO_2_/F_I_O_2_ ≤ 240 and quantitative microbiologic confirmation of pneumonia, the most reliable diagnostic gold-standard. Results: Mean PaO_2_/F_I_O_2_ at VAP onset was 195 ± 82; 171 (67%) cases had PaO_2_/F_I_O_2_ ≤ 240. Patients with PaO_2_/F_I_O_2_ ≤ 240 had a lower APACHE-II score at ICU admission; however, at pneumonia onset they had higher CPIS, SOFA score, acute respiratory distress syndrome criteria and incidence of shock, and less microbiological confirmation of pneumonia (117, 69% vs. 71, 85%, *p* = 0.008), compared to patients with PaO_2_/FIO_2_ > 240. In multivariate logistic regression, PaO_2_/FIO_2_ ≤ 240 was independently associated with less microbiological confirmation (adjusted odds-ratio 0.37, 95% confidence interval 0.15–0.89, *p* = 0.027). The association between PaO_2_/F_I_O_2_ and microbiological confirmation of VAP was poor, with an area under the ROC curve 0.645. Initial non-response to treatment and length of stay were similar between both groups, while hospital mortality was higher in patients with PaO_2_/F_I_O_2_ ≤ 240. Conclusion: Adding PaO_2_/F_I_O_2_ ratio ≤ 240 to the clinical and radiographic criteria does not help in the diagnosis of VAP. PaO_2_/F_I_O_2_ ratio > 240 does not exclude this infection. Using this threshold may underestimate the incidence of VAP.

## 1. Introduction

Ventilator-associated pneumonia (VAP) remains the most frequent intensive care unit (ICU)-acquired infection [1,2]. Its incidence ranges from 9% to 27% of ventilated patients depending on diagnostic criteria [3]. VAP is associated with an increased length of stay, mortality and health care costs [4,5]. 

The diagnosis of VAP may be complex. Therefore, multiple efforts have been directed to overcome these difficulties and to identify the most appropriate diagnostic strategies [6], including integrative scoring systems that take into account multiple clinical data and microbiological findings. 

The Clinical Pulmonary Infection Score (CPIS) score was developed to objectively diagnose VAP [6]. The score combines six variables: body temperature, white blood cell count, quantity and purulence of tracheal secretions, chest radiograph, bacterial growth in tracheal secretions and oxygenation (PaO_2_/F_I_O_2_ ≤ 240 in mmHg). However, the reported poor sensitivity and specificity of CPIS score in most studies preclude its use as an accurate diagnostic strategy [7,8]. Similarly, the Centers for Disease Control (CDC) and Prevention have proposed the inclusion of a decline in oxygenation, assessed by a PaO_2_/F_I_O_2_ ratio ≤ 240, in the diagnostic criteria for VAP [9]. Current American guidelines define pneumonia as the presence of a new or progressive radiological lung infiltrate plus the clinical evidence that the infiltrate is of an infectious origin and a decline in oxygenation [2]. Indeed, the most reliable evidence of an infectious origin in patients suspected of having VAP combines these criteria with the microbiological identification of the potentially-pathogenic microorganisms (PPM) that cause pneumonia [10]. 

To our knowledge, no studies have comprehensively evaluated the relationship between microbiological diagnosis in populations with clinical suspicion of VAP and better or worse oxygenation, as assessed by the PaO_2_/F_I_O_2_ ratio. 

We hypothesized that patients with worse oxygenation would have more frequent microbiological confirmation of VAP. In order to assess the adequacy of PaO_2_/F_I_O_2_ ≤ 240 to help in the diagnosis of VAP, we compared the characteristics and outcomes of a real-life ICU population with suspected VAP and PaO_2_/F_I_O_2_ ≤ or > 240, with special emphasis on microbiological confirmation.

## 2. Methods (Extended Information is Provided in Supplementary Materials)

### 2.1. Study Population

The study was conducted in six medical and surgical ICUs, overall comprising of 45 beds, at an 800-bed university hospital. Data were prospectively collected from 2007 to 2017. Investigators made daily rounds of each ICU. Patients older than 18 years, mechanically-ventilated for 48 h or more, with suspected VAP, were consecutively included into the study and only the first episode was analyzed. We excluded patients with severe immune-suppression [11]. The institution’s Internal Review Board approved the study (*Comitè Ètic d’Investigació Clínica*, registry number 2009/5427) and written informed consent was obtained from patients or their next-of-kin.

### 2.2. Definition of Pneumonia, Microbiologic Processing, and Antimicrobial Treatment 

The clinical suspicion of pneumonia was based on clinical criteria (new or progressive radiological pulmonary infiltrate together with at least two of the following: (1) temperature >38 °C or < 36 °C; (2) leukocytosis > 12,000/mm^3^ or leucopoenia < 4000/mm^3^; or (3) purulent respiratory secretions) [12,13].

The microbiologic evaluation included the collection of at least one lower respiratory airways sample: tracheobronchial aspirates (TBAS) and/or bronchoscopic [14] or blind bronchoalveolar lavage (BAL) [15] if possible, within the first 24 hours of inclusion [16]. The same sampling method was performed on the third day if clinically indicated. Blood cultures and cultures from the pleural fluid were also collected if clinically justified.

Microbiologic confirmation of pneumonia was defined by the presence of at least one PPM [17] in the respiratory samples above pre-defined values (BAL > 10^4^ and TBAS > 10^5^ colony-forming units/mL, respectively, or any value if the patient was under antibiotic treatment), in pleural fluid or in blood cultures if an alternative cause of bacteremia was ruled out [18,19]. Microbiologic identification and susceptibility testing were performed by standard methods [20]. 

The initial empiric antimicrobial treatment was administered in all patients according to the local adaptation of guidelines [1,21], and subsequently revised according to the microbiologic results. 

The empiric antimicrobial treatment was considered appropriate when the isolated pathogens were susceptible in vitro to at least one of the antimicrobials administrated at adequate dose [22].

The initial response to treatment was evaluated after 72 hours of antimicrobial treatment. In patients with initial non-response to treatment [23,24], respiratory samples and blood cultures were obtained again, and the empiric antimicrobial treatment was revised.

### 2.3. Assessment of the Systemic Inflammatory Response

We evaluated the serum levels of interleukin (IL)-6, IL-8, IL-10, tumour necrosis factor-alpha (TNF-alpha), C-reactive protein (CRP), Procalcitonin (PCT), and mid-regional pro-adrenomedullin (MR-proADM) within the first 24 hours and the third day after the diagnosis of pneumonia. All methods for this analysis have been described in detail elsewhere [25,26]. 

### 2.4. Data Collection

All relevant data were collected at admission and at onset of pneumonia from the medical records and bedside flow charts. Patients were followed until hospital discharge, death or up to 90-days after the diagnosis of pneumonia. Septic shock [27] and acute respiratory distress syndrome (ARDS) [28] were defined according to the previously described criteria. 

### 2.5. Outcomes Variables

The rate of quantitative microbiological confirmation of patients with PaO_2_/F_I_O_2_ ≤ 240 was compared with that of patients with PaO_2_/F_I_O_2_ > 240. The lowest value of PaO_2_/FiO_2_ on the day of VAP diagnosis was considered for patients’ group allocation. Secondary outcomes were length of stay and mortality, and 90-day survival after VAP diagnosis. 

### 2.6. Statistical Analysis 

Categorical and continuous data are presented as number (percentage) and as mean ± SD (or median, inter-quartile range), respectively. Categorical variables were compared with the Chi-square or Fisher’s exact tests. Quantitative continuous variables were compared using the unpaired Student’s *t*-test or the Mann-Whitney test for normally and not normally distributed variables, respectively. The Kaplan-Meier curves were used to compare survival in the two groups. 

Univariate and multivariate logistic regression analyses were performed to assess the association of PaO_2_/F_I_O_2_ ≤ 240 or > 240 with positive microbiology. Variables that showed a significant result univariately (*p* < 0.10) were included in the corresponding multivariate logistic regression backward stepwise model. Adjusted odds-ratio (OR) and 95% confidence intervals (CI) were calculated. 

In addition, the association between PaO_2_/F_I_O_2_ at VAP onset as continuous variable and quantitative microbiological confirmation was assessed using receiver-operator-characteristics (ROC) curve analysis. The area under the ROC curve (AUC) with 95% confidence interval (CI), and the optimal cut-off value of PaO_2_/F_I_O_2_ with sensitivity and specificity were calculated.

A two-sided *p*-value ≤ 0.05 was considered statistically significant. All statistical analyses were performed using SPSS 22.0.0.0 (Chicago, IL, USA). 

## 3. Results

### 3.1. Patients’ Characteristics

**Figure 1:**
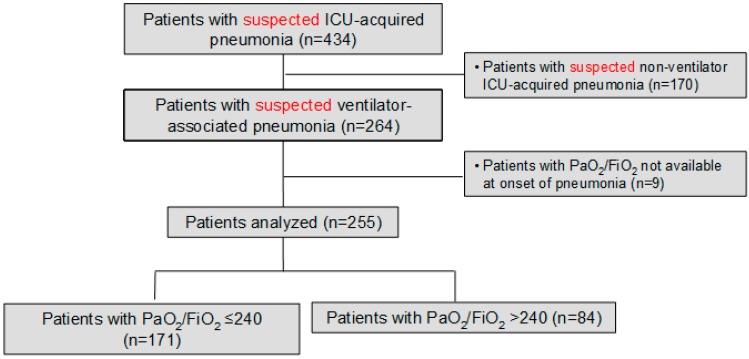
Flow chart of the study population.

We prospectively identified 264 consecutive patients with suspected VAP during the study period; we excluded nine cases because PaO_2_/FiO_2_ at onset of pneumonia was not available. Therefore, we included 255; PaO_2_/FiO_2_ was ≤240 in 171 (67%) patients, and higher than this value in 84 (33%) patients (Figure 1). 

The characteristics of patients at ICU admission and pneumonia onset according PaO_2_/F_I_O_2_ are summarized in Table 1 and Table 2. 

**Figure 2:**
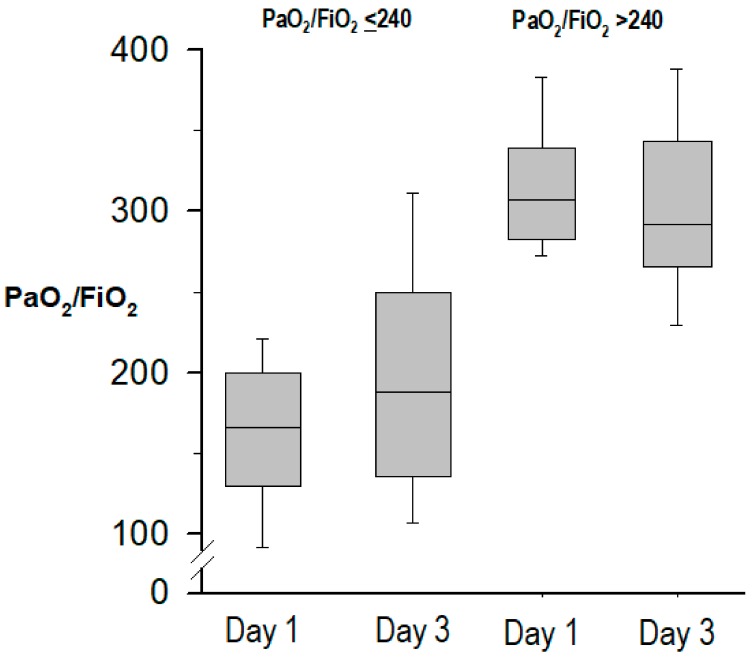
Evolution of PaO_2_/FiO_2_ from pneumonia onset (day 1) to day 3.

Compared to patients with PaO_2_/FIO_2_ > 240, those with PaO_2_/FIO_2_ ≤ 240 had a lower APACHE-II score at ICU admission. However, at onset of pneumonia, patients with PaO_2_/FIO_2_ ≤ 240 had a higher SOFA score and CPIS, in both cases due to the lower values of PaO_2_/FIO_2_, presented ARDS criteria and shock more frequently, and had lower serum levels of sodium. Reasons for ICU admission were slightly different between groups (*p* = 0.051), with higher proportions of multiple trauma and septic shock among patients with PaO_2_/FIO_2_ > 240, and higher proportions of postoperative care and hypoxemic respiratory failure in patients with PaO_2_/FIO_2_ ≤ 240. The evolution of PaO2/FiO_2_ at pneumonia onset and at day 3 in both groups is shown in Figure 2.

### 3.2. Microbiological Aetiology

The etiologic diagnosis of patients is shown in Table 3. The number of samples processed for microbiology was similar between both groups. Pneumonia was microbiologically confirmed in 188 (74%) cases, with a lower rate of microbiological confirmation in patients with PaO_2_/FIO_2_ ≤ 240, compared with those with PaO_2_/FIO_2_ > 240 (117, 69% vs. 71, 85%, *p* = 0.007; odds-ratio 0.40, 95% CI 0.21 to 0.79, *p* = 0.008). The multivariate logistic regression analysis showed that PaO_2_/FIO_2_ ≤ 240 mmHg was independently associated with less microbiological confirmation of pneumonia (adjusted OR 0.37, 95% CI 0.15–0.89, *p* = 0.027). The association between PaO_2_/F_I_O_2_ and microbiological confirmation of VAP was poor, although statistically significant, with an AUC 0.645 (95% CI 0.568 to 0.725, *p* = 0.040). The optimal cut-off value of PaO_2_/FIO_2_ associated with microbiological confirmation of VAP was 223, with sensitivity 45% and specificity 79%.

The most frequent pathogens identified were *Pseudomonas aeruginosa*, Gram-negative enteric bacteria and *Staphylococcus aureus*, with no significant differences in the relative proportion of any pathogen among patients with microbiological diagnosis of ICUAP. 

### 3.3. Systemic Inflammatory Response

The serum levels of all inflammatory biomarkers at pneumonia onset and at day 3 are shown in Table 4. Patients with PaO_2_/FIO_2_ ≤ 240 had higher serum levels of Procalcitonin at pneumonia onset, without any significant difference in the remaining biomarkers.

### 3.4. Empiric Antibiotic Treatment and Outcome Variables 

Initial non-response to treatment, length of stay and ICU mortality were similar between both groups (Table 5). 

**Figure 3:**
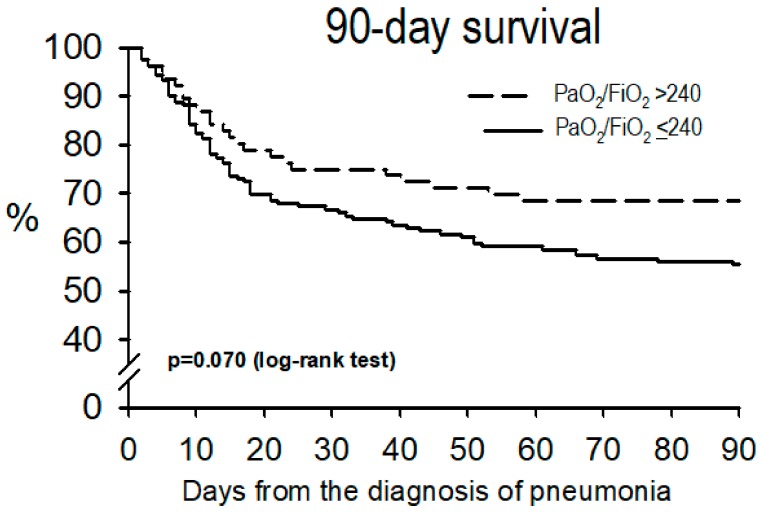
Survival curves at 90 days.

Hospital mortality was higher, and the ventilator-free days were lower, in patients with PaO_2_/F_I_O_2_ ≤ 240, while 90-day survival tended to be lower in this group (*p* = 0.070, Figure 3). 

## 4. Discussion

To the best of our knowledge, this is the first study investigating the relationship between quantitative microbiological confirmation and oxygenation in a prospective cohort of patients mechanically ventilated and suspected of having VAP. We found that PaO_2_/F_I_O_2_ ≤ 240 was associated with less microbiological confirmation in this subgroup of patients. In addition, patients with worse oxygenation had higher hospital mortality.

The diagnosis of VAP yields on clinical criteria; however, due to their potential subjectivity, other parameters has been proposed in order to better define VAP episodes [29]. Over the last years, due to an excess of antibiotic treatment, the United States Critical Care Collaborative Societies and the Department of Health and Human Services have proposed the implementation of “ventilator-associated complications” and “ventilator-associated infections” [30]. However, the use of “more objective” criteria such as chest X-ray and oxygenation has important limitations. Chest X-rays may be difficult to interpret in ICU patients due to multiple non-infectious causes such as congestive heart failure, atelectasis, prior chronic lung disease, or ARDS. 

Microbiological confirmation is probably the best matching to assess VAP diagnosis quality [10]. We hypothesized that patients with more impaired oxygenation would have more frequently etiologic diagnosis and therefore they would be considered as definitive VAP episodes. Surprisingly our findings demonstrated the opposite. We previously reported that negative microbiology in patients with both community-acquired and ICU-acquired pneumonia was associated with renal and cardiac co-morbidities [31,32]. These studies suggested that some of these cases might also represent, at least in part, fluid overload because of renal failure or congestive heart failure added to the underlying inflammatory process potentially mimicking pneumonia. These conditions often exhibit more severe deterioration of oxygenation, thus possibly explaining the association of lower PaO_2_/F_I_O_2_ with less microbiological confirmation of VAP. However, lack of detection of a PPM would not preclude existence of an infection since many patients are previously treated with antibiotics. Therefore, until more specific diagnostic criteria are available, initiation of antimicrobial treatment should still be based on the clinical signs of infection together with radiographic criteria. 

The use of oxygenation has been proposed in many diseases as a criterion for diagnosis. Indeed, in case of ARDS PaO_2_/F_I_O_2_ ratio is both a diagnostic and stratification tool [28,33]. Therefore, since pneumonia is the most frequent cause of ARDS [33], PaO_2_/F_I_O_2_ ratio has also been considered for pneumonia diagnosis too. Unfortunately, although PaO_2_/F_I_O_2_ might be a good parameter of severity, it might lead clinicians to over treat patients. In many situations of daily clinical practice, the use of PaO_2_/F_I_O_2_ ratio might trigger antibiotics start when they are probably not needed, and conversely patients with clear infiltrates in the X-rays but without major oxygenation derangements might preclude that the patient is not developing a VAP. In our population, 33% of suspected VAP episodes had a PaO_2_/F_I_O_2_ >240. Therefore, the use of this threshold of PaO_2_/F_I_O_2_ in the diagnosis probably would have underestimated the incidence of VAP. This has important implications, either in the development of antimicrobial resistance due to unnecessary treatment, and an increase in mortality due to inappropriate (or none) antibiotic treatment. Using PaO_2_/F_I_O_2_ may be important to stratify patients’ severity in controlled clinical trials, and probably for an earlier or more aggressive treatment, as we report higher hospital mortality for those patients with worse oxygenation. We think, however, that patients with higher PaO_2_/F_I_O_2_ should not be excluded from clinical trials dealing with VAP.

One probable reason to point out oxygenation as a diagnosis marker is due to a wrong interpretation of CPIS as a clinical marker for VAP [6]. The CPIS can be a useful tool for severity identification when the patient has been diagnosed of VAP and can help to address patients’ response to treatment. Luna et al. using PaO_2_/FIO_2_ ratio could demonstrate that this was the best parameter for patients’ stratification of treatment response and a marker of clinical cure [34]. Moreover, we have previously reported that lack of improvement of PaO_2_/FIO_2_ was among the best predictors for adverse outcomes in ICU-acquired pneumonia [24]. This, however, faces against the use of PaO_2_/F_I_O_2_ as a diagnostic criterion, especially in some subsets of patients such as those with multiple trauma, surgery, or major burns [8]. Indeed, we found in the present study that patients with postoperative VAP episodes presented more hypoxemia compared to patients with multiple trauma. On the other hand, the recent HAP/VAP International guidelines [35] made a weak recommendation for short antibiotic courses in patients with low CPIS (i.e., 6 or less). However, this does not seem to be related to diagnosis purposes. 

Diagnosis of VAP including oxygenation has not been universally accepted by many authors. In a European cohort study conducted in 465 patients with VAP, worsening oxygenation was present in 3 out of 4 patients [36], whilst Martin-Loeches et al. found it in two-thirds of their patients with VAP [37]. Similar to severe CAP [38], oxygenation can be and probably should be taken as a severity criteria similar to septic shock development, but it should not substitute current diagnostic parameters widely used. Several studies performed over the last decade have found that prompt antibiotic therapy was the most important determinant for improved outcomes in a general sepsis population [39]. It does not seem reasonable to add a test that based on our results would delay and probably mislead VAP diagnosis.

The current study has important strengths. It includes a long cohort of patients prospectively collected and without significant loss of follow-up due to a careful surveillance with a dedicated research team. In all the patients, blood gases were taken at the moment of diagnosis based on local protocols. Because of inaccuracy to assess the actual FiO_2_ in non-intubated patients, we did not include cases of non-ventilator ICU-acquired pneumonia [40]. Moreover, all patients had at least one lower respiratory tract sample processed for culture at onset of pneumonia, and the frequency and type of previous antibiotic treatment is reported, without differences related to severity of oxygenation in both variables. An additional value for this study is the acquisition of inflammatory markers at the onset of pneumonia and after three days of antibiotic treatment. However, we have to acknowledge some limitations. The most important would be the single centre analysis, which might limit reproducibility of the results. However, since the same research team worked on the project during the inclusion period, the study appears reliable in terms of diagnosis and homogeneity. Second, we did not collect blood gas data at ICU admission and therefore cannot assess the evolution of oxygenation before patients were suspected of having VAP.

## 5. Conclusions

In conclusion, we have found that adding PaO_2_/F_I_O_2_ ratio ≤ 240 to the clinical and radiographic criteria does not help in the diagnosis of VAP. PaO_2_/F_I_O_2_ ratio > 240 does not exclude this infection. Using this threshold may underestimate the incidence of VAP. Changing this paradigm might have beneficial effects on the early antibiotic treatment for VAP.

## Figures and Tables

**Table 1:** Baseline characteristics of patients at ICU admission.

	PaO_2_/F_I_O_2_ ≤ 240 *n* = 171	PaO_2_/F_I_O_2_ > 240 *n* = 84	*p* Value
Age, year	62 ± 16	61 ± 15	0.57
Sex, male/female, *n*	116/55	60/24	0.56
Alcohol abuse (current or former), *n* (%)	41 (24)	18 (21)	0.63
Smoking habit (current or former), *n* (%)	90 (53)	40 (48)	0.45
APACHE-II score	17 ± 6	19 ± 8	0.044
SAPS score	42 ± 15	45 ± 16	0.24
SOFA score	8 ± 3	8 ± 3	0.71
Co-morbidities, *n* (%)			
Diabetes mellitus	41 (24)	19 (23)	0.81
Chronic renal failure	15 (9)	5 (6)	0.43
Solid cancer	19 (11)	10 (12)	0.85
Chronic heart disorders	54 (32)	28 (33)	0.78
Chronic lung disease	58 (34)	30 (24)	0.10
Chronic liver disease	27 (16)	9 (11)	0.27
Recent surgery, *n* (%)	81 (47)	43 (51)	0.57
Tracheotomy at admission, *n* (%)	16 (9)	11 (13)	0.37
Causes of ICU admission, *n* (%)			0.051
Post-operative	40 (23)	13 (16)	
Hypoxemic respiratory failure	20 (12)	5 (6)	
Decreased consciousness	31 (18)	17 (20)	
Hypercapnic respiratory failure	16 (9)	5 (6)	
Septic shock	12 (7)	10 (12)	
Multiple trauma	12 (7)	16 (19)	
Non-surgical abdominal disease	5 (3)	3 (4)	
Acute coronary syndrome	10 (6)	2 (2)	
Cardiac arrest	12 (7)	9 (11)	
Other	6 (4)	4 (5)	

Definition of abbreviations: APACHE: Acute Physiology and Chronic Health Evaluation; ICU: Intensive Care Unit; SAPS: Simplified Acute Physiology Score; SOFA: Sepsis-related Organ Failure Assessment.

**Table 2:** Characteristics of patients at pneumonia onset.

	PaO_2_/F_I_O_2_ ≤ 240 *n* = 171	PaO_2_/F_I_O_2_ > 240 *n* = 84	*p* Value
Previous antibiotics, *n* (%)	139 (81)	66 (79)	0.61
Most frequent groups, *n* (%):			
Penicillins	78 (46)	26 (31)	
Cephalosporins	50 (29)	26 (31)	
Quinolones	48 (28)	20 (24)	
Carbapenems	23 (13)	14 (17)	
Glycopeptides	28 (16)	9 (11)	
Aminoglycosides	15 (9)	11 (13)	
Clindamycin	14 (8)	11 (13)	
Antifungals	6 (4)	2 (2)	
Hospital stay before pneumonia, days *	7 (4–13)	7 (5–15)	0.34
ICU stay before pneumonia, days *	5 (3–9)	6 (4–10)	0.12
SOFA score	8 ± 3	7 ± 3	0.004
Bilateral pulmonary infiltrates, *n* (%)	55 (32)	19 (23)	0.11
ARDS criteria, *n* (%)	32 (19)	1 (1)	<0.001
Pleural effusion, *n* (%)	56 (33)	18 (22)	0.10
PaO_2_/FiO_2_	161 ± 47	301 ± 49	<0.001
FiO_2_	0.55 ± 0.18	0.41 ± 11	<0.001
PEEP, cmH_2_O	7.4 ±3.4	7.8 ± 3.0	0.36
PaCO_2_, mmHg	41 ± 9	39 ± 7	0.066
pHa	7.40 ± 0.09	7.41 ± 0.07	0.33
Shock at onset of pneumonia, *n* (%)	96 (56)	32 (38)	0.007
Temperature	36.9 ± 1.4	37.0 ± 1.3	0.37
Serum creatinine, mg/dL	1.3 ± 1.0	1.2 ± 1.2	0.85
Blood haemoglobin, g/L	11 ± 2	10 ± 2	0.67
White blood cell count, L^−9^	14 ± 7	13 ± 6	0.11
Sodium, mEq/L	139 ± 6	141 ± 8	0.042
Potassium, mEq/L	4.0 ± 0.7	4.1 ± 0.5	0.75
CPIS day 1	7 ± 1	5 ± 1	<0.001
CPIS day 3	6 ± 2	5 ± 2	<0.001

* Results given as median (inter-quartile range). Definition of abbreviations: ICU: Intensive Care Unit; SOFA: Sepsis-related Organ Failure Assessment; ARDS: Acute Respiratory Distress Syndrome; PaO_2_/FiO_2:_ ratio of arterial oxygen tension to inspired oxygen fraction; CPIS: Clinical Pulmonary Infection Score.

**Table 3:** Etiologic diagnosis of pneumonia according to PaO_2_/F_I_O_2._

Pathogen	PaO_2_/F_I_O_2_ ≤ 240 *n* = 171	PaO_2_/F_I_O_2_ > 240 *n* = 84	*p* Value
Sample processed for microbiology, *n* (%)			
Endotracheal aspirates	157 (92)	79 (94)	0.52
Bonchoalveolar lavage	38 (23)	21 (25)	0.62
Blood	126 (74)	58 (69)	0.44
Pleural fluid	18 (11)	5 (6)	0.23
Positive microbiology, *n* (%)	117 (69)	71 (85)	0.007
Gram-positive bacteria, *n* (%)	37 (32)	23 (32)	0.96
MS *Staphylococcus aureus*	18 (15)	17 (24)	0.20
MR *Staphylococcus aureus*	12 (10)	4 (6)	0.41
*Streptococcus pneumoniae*	8 (7)	2 (3)	0.39
Gram-negative enteric bacteria, *n* (%)	39 (33)	16 (23)	0.16
*Enterobacter* spp	7 (6)	2 (3)	0.53
*Klebsiella* spp	11 (9)	6 (9)	0.78
*Escherichia coli*	9 (8)	2 (3)	0.62
*Proteus* spp	2 (2)	2 (3)	0.99
*Citrobacter* spp	3 (3)	3 (4)	0.84
*Serratia* spp	8 (7)	4 (6)	0.97
*Morganella morganii*	1 (1)	0 (0)	0.80
Non-fermentating gram-negative bacilli	38 (32)	29 (41)	0.32
*Stenotrophomonas maltophilia*	7 (6)	3 (4)	0.85
*Pseudomonas aeruginosa*	31 (27)	26 (37)	0.19
Other gram-negative bacteria			
*Moraxella catarrhalis*	2 (2)	0 (0)	0.71
*Haemophilus influenzae*	3 (3)	4 (6)	0.50
Fungi, *n* (%)	2 (2)	1 (1)	0.66
*Aspergillus* spp	2 (2)	1 (1)	0.66
Others, *n* (%)	0 (0)	1 (5)	-
Polymicrobial aetiology *	19 (11)	9 (11)	0.91

* Polymicrobial pneumonia was defined when more than one potentially-pathogenic microorganism was identified as causative agents.

**Table 4:** Serum levels of inflammatory biomarkers *.

	*n* ^†^	PaO_2_/F_I_O_2_ ≤ 240 *n* = 171	*n* ^†^	PaO_2_/F_I_O_2_ > 240 *n* = 84	*p* Value
C-reactive protein day 1, mg/dL	164	13 (6–21)	81	11 (4–19)	0.13
C-reactive protein day 3, mg/dL	149	11 (4–19)	78	9 (5–15)	0.078
IL-6 day 1, pg/mL	89	168 (72–431)	36	109 (41–229)	0.077
IL-6 day 3, pg/mL	75	91 (19–204)	32	78 (33–183)	0.98
IL-8 day 1, pg/mL	89	108 (64–214)	36	94 (57–137)	0.17
IL-8 day 3, pg/mL	75	76 (41–145)	32	99 (63–170)	0.15
TNF-alpha day 1, pg/mL	89	8 (5–17)	36	7 (5–14)	0.73
TNF-alpha day 3, pg/mL	75	7 (4–14)	32	8 (5–15)	0.61
Procalcitonin day 1, ng/mL	90	0.45 (0.14–1.37)	37	0.19 (0.07–0.59)	0.037
Procalcitonin day 3, ng/mL	78	0.30 (0.10–1.06)	34	0.20 (0.08–0.74)	0.57
MR-proADM day 1, nmol/L	98	1.27 (0.33–2.22)	40	1.16 (0.65–2.23)	0.84
MR-proADM day 3, nmol/L	87	1.36 (0.38–2.37)	37	1.08 (0.61–2.04)	0.92

* Results are given as median (inter-quartile range). ^†^ Number of cases with blood samples processed for each inflammatory biomarker and group. Definition of abbreviations: IL: interleukin; TNF: tumor necrosis factor; MR-proADM: mid-regional pro-adrenomedullin.

**Table 5:** Outcome variables.

	PaO_2_/F_I_O_2_ ≤ 240 *n* = 171	PaO_2_/F_I_O_2_ > 240 *n* = 84	*p* Value
ICU stay, days *	19 (12–29)	18 (13–32)	0.75
Hospital stay, days *	34 (19–56)	43 (21–56)	0.34
Non-response to treatment, *n* (%)	94 (55)	48 (57)	0.74
ICU mortality, *n* (%)	53 (31)	18 (24)	0.11
Hospital mortality, *n* (%)	71 (42)	24 (29)	0.044
Ventilator-free days at day 28 *	3 (0–21)	17 (0–24)	0.022
Causes of death within 90 days:			0.28
Shock, *n* (%)	52 (74)	14 (58)	
Refractory hypoxemia, *n* (%)	4 (6)	2 (8)	
Order do-not-resuscitate, *n* (%)	1 (1)	2 (8)	
Brain anoxia, *n* (%)	11 (16)	6 (25)	
Others, *n* (%)	2 (3)	0 (0)	

* Results given as median (inter-quartile range). Definition of abbreviations: ICU: Intensive Care Unit.

## Supplementary Materials

The following are available online at https://www.mdpi.com/2077-0383/8/8/1217/s1, Supplementary Data, Methods.
