# Supplementary material for: Ventilator-Associated Pneumonia and PaO2/FIO2 Diagnostic Accuracy: Changing the Paradigm?
Source: J Clin Med. 2019 Aug 14;8(8):1217. doi: 10.3390/jcm8081217 (PMC6722826; doi:10.3390/jcm8081217)
Supplement: Supplementary file 1 [file jcm-08-01217-s001.pdf]

Supplementary Material

Extended Methods

Study Population

The study was conducted in six medical and surgical ICUs, overall comprising 45 beds, at an 800-bed university hospital. Data were prospectively collected from 2007 to 2017. Investigators made daily rounds of each ICU. Patients older than 18 years, mechanically-ventilated for 48 hours or more, with clinical suspicion of VAP were consecutively included in the study and only the first episode was analyzed. We excluded patients with severe immune-suppression (neutropenia after chemotherapy or hematopoietic transplant, drug-induced immune-suppression in solid-organ transplant or cytotoxic therapy, and human immunodeficiency virus infected patients) [1]. The institution's Internal Review Board approved the study (*Comitè Ètic d'Investigació Clínica*, registry number 2009/5427) and written informed consent was obtained from patients or their next-of-kin.

Definition of Pneumonia, Microbiologic Processing, and Antimicrobial Treatment

The clinical suspicion of pneumonia was based on clinical criteria (new or progressive radiological pulmonary infiltrate together with at least two of the following: (1) temperature >38 °C or <36 °C; (2) leukocytosis >12,000/mm^3 or leucopoenia <4,000/mm^3; or (3) purulent respiratory secretions) [2,3].

The microbiologic evaluation included the collection of at least one lower respiratory airways sample: tracheobronchial aspirates (TBAS) and/or bronchoscopic [4] or blind bronchoalveolar lavage (BAL) [5], if possible within the first 24 hours of inclusion [6]. Only samples of tracheal aspirates of high quality (i.e. <10 squamous cells and >25 leukocytes per optical microscopy field) were processed for culture. The same sampling method was performed on the third day if clinically indicated. Blood cultures and cultures from the pleural fluid were also collected if clinically justified. Urinary antigens of *Streptococcus pneumoniae* and *Legionella pneumophila* were not systematically collected.

Microbiologic confirmation of pneumonia was defined by the presence of at least one PPM, i.e. endogenous microorganisms associated with nosocomial respiratory infection in the host [7], in the respiratory samples with above pre-defined values (BAL > 10^4 and TBAS > 10^5 colony-forming units/ml, respectively, or any value if the patient was under antibiotic treatment), in pleural fluid or in blood cultures if an alternative cause of bacteremia was ruled out [8,9]. Microbiologic

identification and susceptibility testing were performed by standard methods [10]. Polymicrobial pneumonia was defined when more than one PPM were identified as causative agents.

The initial empiric antimicrobial treatment was administered to all patients according to local adaptation of American Thoracic Society (ATS)/Infectious Disease Society of America (IDSA) guidelines [11,12], based on the most frequently isolated PPM and their patterns of antimicrobial sensitivity in our institution, and subsequently revised according to the microbiologic results.

The empiric antimicrobial treatment was considered appropriate when the isolated pathogens were susceptible in vitro to at least one of the antimicrobials administrated at adequate dose [13].

The initial response to treatment was evaluated after 72 hours of antimicrobial treatment. Non-response to treatment was defined by the presence of at least one of the following criteria: No improvement of the arterial PaO_2/FiO_2; need for intubation because of pneumonia (defined as need for intubation after 24 h from the beginning of antibiotics); persistence of fever (temperature ≥ 38 °C) or hypothermia (<35.5 °C) together with purulent respiratory secretions; increase in pulmonary infiltrates on chest radiograph ≥ 50 %; occurrence of septic shock or multiple organ dysfunction syndrome, defined as three or more organ system failures not present on day one. In patients with initial non-response to treatment [14,15], respiratory samples and blood cultures were obtained again, and the empiric antimicrobial treatment was revised.

Assessment of the Systemic Inflammatory Response

We evaluated the serum levels of interleukin (IL)-6, IL-8, IL-10, tumor necrosis factor-alpha (TNF-alpha), C-reactive protein (CRP) and Procalcitonin (PCT) within the first 24 hours and the third day after the diagnosis of pneumonia. We also determined mid-regional pro-adrenomedullin (MR-proADM) using a test based on the time-resolved amplified cryptate emission technology (KRYPTOR; BRAHMS AG; Hennigsdorf, Germany). MR-proADM has an analytical detection limit of 0.08 nmol/L (normal reference range 0.33 ± 0.7 nmol/L) and a functional assay sensitivity of 0.12 nmol/L. All methods for this analysis have been described in detail elsewhere [16,17].

Data Collection

All relevant data were collected at admission and at onset of pneumonia from the medical records and bedside flow charts, including laboratory, radiologic and microbiologic information. We calculated the Acute Physiology And Chronic Health Evaluation (APACHE-II) [18], the Simplified Acute Physiology Score (SAPS) [19], and the Sepsis-related Organ Failure Assessment (SOFA) [20] scores. Patients were followed until hospital discharge, death or up to 90 days after

the diagnosis of pneumonia. Septic shock [21] and acute respiratory distress syndrome (ARDS) [22] were defined according to the previously described criteria.

Outcomes Variables

The rate of microbiological confirmation of patients with $PaO_2/F_IO_2 \leq 240$ was compared with that of patients with $PaO_2/F_IO_2 > 240$ mmHg. The lowest value of PaO_2/FiO_2 on the day of VAP diagnosis was considered for patients' group allocation. Secondary outcomes were ICU and hospital length of stay and mortality, and survival at 90 days after VAP diagnosis.

Statistical analysis

Categorical and continuous data are presented as number (percentage) and as mean±SD (or median, inter-quartile range), respectively. Categorical variables were compared with the Chi-square or Fisher's exact tests. Quantitative continuous variables were compared using the unpaired Student's t-test or the Mann-Whitney test for normally and not-normally distributed variables, respectively. The Kaplan-Meier curves were used to compare survival in the two groups.

Univariate and multivariate logistic regression analyses were performed to assess the association of $PaO_2/F_IO_2 \leq 240$ or > 240 with positive microbiology. Variables that showed a significant result univariately ($p < 0.10$) were included in the corresponding multivariate logistic regression backward stepwise model. Adjusted odds-ratio (OR) and 95% confidence intervals (CI) were calculated.

In addition, the association between PaO_2/F_IO_2 at VAP onset as continuous variable and quantitative microbiological confirmation was assessed using receiver-operator-characteristics (ROC) curve analysis. The area under the ROC curve (AUC) with 95% confidence interval (CI), and optimal cut-off value of PaO_2/F_IO_2 with sensitivity and specificity were calculated.

A two-sided p-value ≤ 0.05 was considered statistically significant. All statistical analyses were performed using SPSS 22.0.0.0 (Chicago, Il).

References

1. Hilbert G, Gruson D, Vargas F, Valentino R, Gbikpi-Benissan G, Dupon M, Reiffers J, Cardinaud JP. Noninvasive ventilation in immunosuppressed patients with pulmonary infiltrates, fever, and acute respiratory failure. *N. Engl. J. Med.* 2001; 344: 481–487.

2. Fàbregas N, Ewig S, Torres A, El-Ebiary M, Ramirez J, de La Bellacasa JP, Bauer T, Cabello H. Clinical diagnosis of ventilator associated pneumonia revisited: comparative validation using immediate post-mortem lung biopsies. *Thorax* 1999; 54: 867–873.

3. Woodhead, M; Torres, A. Definition and classification of community-acquired and nosocomial pneumonias. European Respiratory Society Journals Ltd; 1997.

4. Meduri GU, Chastre J. The standardization of bronchoscopic techniques for ventilator-associated pneumonia. *Chest* 1992; 102: 557S-564S.

5. Kollef MH, Bock KR, Richards RD, Hearns ML. The safety and diagnostic accuracy of minibronchoalveolar lavage in patients with suspected ventilator-associated pneumonia. *Ann. Intern. Med.* 1995; 122: 743–748.

6. Ruiz M, Torres A, Ewig S, Marcos MA, Alcón A, Lledó R, Asenjo MA, Maldonaldo A. Noninvasive versus invasive microbial investigation in ventilator-associated pneumonia: evaluation of outcome. *Am. J. Respir. Crit. Care Med.* 2000; 162: 119–125.

7. van Saene HKF, Petros AJ, Ramsay G, Baxby D. All great truths are iconoclastic: selective decontamination of the digestive tract moves from heresy to level 1 truth. *Intensive Care Med* 2003; 29: 677–690.

8. Ioanas M, Cavalcanti M, Ferrer M, Valencia M, Agusti C, Puig de la Bellacasa J, Torres A. Hospital-acquired pneumonia: coverage and treatment adequacy of current guidelines. *Eur. Respir. J.* 2003; 22: 876–882.

9. Valencia Arango M, Torres Martí A, Insausti Ordeñana J, Alvarez Lerma F, Carrasco Joaquinet N, Herranz Casado M, Tirapu León JP, Grupo de Estudio de la Neumonía Relacionada con Ventilación Mecánica, Grupo de Trabajo de Enfermedades Infecciosas de la SEMICYUC. [Diagnostic value of quantitative cultures of endotracheal aspirate in ventilator-associated pneumonia: a multicenter study]. *Arch. Bronconeumol.* 2003; 39: 394–399.

10. Murray PR, Baron EJ, Joergensen JH, Pfaller MA, Yolken RH. Manual of Clinical Microbiology 8th ed. Washington DC: American Society for Microbiology; 2003.

11. Kalil AC, Metersky ML, Klompas M, Muscedere J, Sweeney DA, Palmer LB, Napolitano LM, O'Grady NP, Bartlett JG, Carratalà J, El Solh AA, Ewig S, Fey PD, File TM, Restrepo MI, Roberts JA, Waterer GW, Cruse P, Knight SL, Brozek JL. Management of Adults With Hospital-acquired and Ventilator-associated Pneumonia: 2016 Clinical Practice Guidelines

by the Infectious Diseases Society of America and the American Thoracic Society. *Clin. Infect. Dis.* 2016; 63: e61–e111.

12. American Thoracic Society, Infectious Diseases Society of America. Guidelines for the management of adults with hospital-acquired, ventilator-associated, and healthcare-associated pneumonia. *Am. J. Respir. Crit. Care Med.* 2005; 171: 388–416.

13. Heyland DK, Dodek P, Muscedere J, Day A, Cook D, Canadian Critical Care Trials Group. Randomized trial of combination versus monotherapy for the empiric treatment of suspected ventilator-associated pneumonia. *Crit. Care Med.* 2008; 36: 737–744.

14. Ioanas M, Ferrer M, Cavalcanti M, Ferrer R, Ewig S, Filella X, de la Bellacasa JP, Torres A. Causes and predictors of nonresponse to treatment of intensive care unit-acquired pneumonia. *Crit. Care Med.* 2004; 32: 938–945.

15. Esperatti M, Ferrer M, Giunta V, Ranzani OT, Saucedo LM, Li Bassi G, Blasi F, Rello J, Niederman MS, Torres A. Validation of predictors of adverse outcomes in hospital-acquired pneumonia in the ICU. *Crit. Care Med.* 2013; 41: 2151–2161.

16. Ramírez P, Ferrer M, Martí V, Reyes S, Martínez R, Menéndez R, Ewig S, Torres A. Inflammatory biomarkers and prediction for intensive care unit admission in severe community-acquired pneumonia. *Crit. Care Med.* 2011; 39: 2211–2217.

17. Bello S, Lasierra AB, Mincholé E, Fandos S, Ruiz MA, Vera E, de Pablo F, Ferrer M, Menendez R, Torres A. Prognostic power of proadrenomedullin in community-acquired pneumonia is independent of aetiology. *Eur. Respir. J.* 2012; 39: 1144–1155.

18. Knaus WA, Draper EA, Wagner DP, Zimmerman JE. APACHE II: a severity of disease classification system. *Crit. Care Med.* 1985; 13: 818–829.

19. Le Gall JR, Lemeshow S, Saulnier F. A new Simplified Acute Physiology Score (SAPS II) based on a European/North American multicenter study. *JAMA* 1993; 270: 2957–2963.

20. Vincent JL, Moreno R, Takala J, Willatts S, De Mendonça A, Bruining H, Reinhart CK, Suter PM, Thijs LG. The SOFA (Sepsis-related Organ Failure Assessment) score to describe organ dysfunction/failure. On behalf of the Working Group on Sepsis-Related Problems of the European Society of Intensive Care Medicine. *Intensive Care Med* 1996; 22: 707–710.

21. Singer M, Deutschman CS, Seymour CW, Shankar-Hari M, Annane D, Bauer M, Bellomo R, Bernard GR, Chiche J-D, Coopersmith CM, Hotchkiss RS, Levy MM, Marshall JC,

Martin GS, Opal SM, Rubenfeld GD, van der Poll T, Vincent J-L, Angus DC. The Third International Consensus Definitions for Sepsis and Septic Shock (Sepsis-3). *JAMA* 2016; 315: 801–810.

22. ARDS Definition Task Force, Ranieri VM, Rubenfeld GD, Thompson BT, Ferguson ND, Caldwell E, Fan E, Camporota L, Slutsky AS. Acute respiratory distress syndrome: the Berlin Definition. *JAMA* 2012; 307: 2526–2533.
